# Teaching the Teachers About Language Support Strategies: Effects on Young Children's Language Development

**DOI:** 10.3389/fpsyg.2021.660750

**Published:** 2021-05-04

**Authors:** Katharina Voltmer, Oliver Hormann, Marcus Pietsch, Claudia Maehler, Maria von Salisch

**Affiliations:** ^1^Institute of Psychology, Leuphana University Lueneburg, Lueneburg, Germany; ^2^Institute for Educational Sciences, Technical University Braunschweig, Braunschweig, Germany; ^3^Center for Methods, Leuphana University Lueneburg, Lueneburg, Germany; ^4^Institute of Educational Science, Leuphana University Lueneburg, Lueneburg, Germany; ^5^Institute of Education, University of Zurich, Zurich, Switzerland; ^6^Institute of Psychology, University of Hildesheim, Hildesheim, Germany

**Keywords:** kindergarten, early childhood, teacher-led intervention, language, language support strategies

## Abstract

The feeling thinking talking (FTT) intervention was designed because early childhood seems to be a prime time for fostering young children's language skills. This intervention involved teaching teachers from *N* = 28 kindergarten groups in *N* = 13 German kindergartens language support strategies (LSS) to be used in everyday conversations with the children in their care. The FTT intervention was evaluated in a business-as-usual control group design with *N* = 281 children (mean age = 49.82 months, range = 33-66 months at T1, mixed SES) who were individually tested using objective tests on grammar, vocabulary and working memory before (T1) and after the FTT intervention (T2), and in a follow-up about one year after T1 (T3). After propensity matching was applied, multilevel models demonstrated that the children taught by the intervention group teachers made faster progress in their understanding of sentences, their application of morphological rules, and their memory for sentences when numerous covariates (child age, gender, behavioral self-regulation, multilingual upbringing, and family SES) were controlled. Results suggest that complex language processing abilities in young children can be promoted by a teacher-led intervention in early childhood education. Improved language skills will further all children's academic and social success in school.

Language skills impact on many areas of child development. Being able to verbalize what they think, how they feel, and who they are, and being able to understand others who express and share their inner lives verbally, helps children and adolescents to acquire new concepts (Vygotsky, 1962), regulate their emotions (e.g., Cole et al., 2010; Rose et al., 2018), and form their identities (e.g., Salomone, 2010). Our aim is to present a novel intervention which trains teachers in early childhood education in using language support strategies (LSS) when talking to the children about internal conditions (i.e., emotion talk) and external observations (i.e., scientific reasoning). A second aim is to evaluate the effect of this multifocus intervention on child language development.

Promoting language skills is an important aim in early childhood education: Language skills facilitate mental representation, manipulation, and memorization of learning content (Vygotsky, 1962), verbal co-construction of the meaning of new ideas and embedding them in prior knowledge (e.g., Rogoff, 1998; Leseman et al., 2001; Littleton and Mercer, 2013). A number of studies corroborate the value of early language skills for later academic achievement, especially written language performance (Durham et al., 2007; Lehrl et al., 2020; Stumm et al., 2020). Children with more advanced kindergarten language skills are likely to demonstrate better reading abilities from grades 1 to 3 and 3 to 5 in primary school (Pace et al., 2019).

Large individual differences in children's language comprehension and production tend to correspond to the socio-economic status (SES) of their families (e.g., Rowe, 2018). While parents from high SES families tend to talk more, use a wider vocabulary (Weizman and Snow, 2001), and more complex syntax (Huttenlocher et al., 2010), parents from low SES families tend to talk less about abstract concepts with their young children. Nevertheless, considerable variety exists within SES groups in terms of the quantity and quality of parents' input, which influences many facets of child language development (e.g., Pace et al., 2017; Papura, 2019; Sperry et al., 2019). Language seems to play an important role in transmitting SES-related disadvantages (Stumm et al., 2020). To compensate for these disadvantages, socialization beyond the family context is important and enhancing language development becomes a central objective of early childhood education.

On average, 28% of the children attending preschools and kindergartens (which are not part of formal schooling in Germany) have at least one immigrant parent. About two thirds of all them are dual language learners (DLL) who first come into contact with German in elementary education and care institutions (Autorengruppe Bildungsberichterstattung, 2020). There is an urgent need to improve the language skills, especially in the register of academic language (Volodina et al., 2020), of the DLLs and of all other children whose families provide less stimulating language input to prepare them for formal schooling, which begins around age six in first grade of primary school.

Language interventions in elementary education leverage young children's neuropsychological preparedness for language learning and the neural plasticity in their brains which relates language input to brain structures (Merz et al., 2019). According to Vygotsky (1978), the success of language input depends on social (primarily linguistic) interactions with adults. Therefore, early childhood seems to be a prime time for interventions designed to enhance children's vocabulary and grammar. This will contribute to school readiness for all children.

## Language Support Strategies

One of the core elements of language interventions in early childhood institutions is the use of language support strategies (LSS; Girolametto and Weitzman, 2002). Examples of LSS are listed in Table 1. LSS serve three main purposes: (1) to present frequent, highly-informative linguistic input, (2) to elicit conversation from the children, and (3) to supply them with feedback or new information (e.g., expansions, recasts) contingent on their output, i.e., their speech (Baker and Nelson, 1984; Hoff-Ginsberg, 1986; Crain-Thoreson and Dale, 1999; Vigil et al., 2005; Raver et al., 2012). The first purpose is achieved, e.g., by adult utterances, called *parallel talk*, that attach new vocabulary to actions, thoughts, or feelings the child cannot express, or through utterances that *repeat* part of the child's prior utterance (Hoff-Ginsberg, 1986). The second function is addressed by questions of different kinds. Those who are more *open-ended* contribute to language acquisition by asking the child to produce a complex answer, as compared to *simple wh-questions* that, among other things, prompt the child to reproduce a familiar word. The third function of feedback is fulfilled by utterances that add new information to the child's prior utterance (*expansion*) or repeat it with proper grammar, lexicon etc. (*indirect correction*).

**Table 1:** Examples of language support strategies.

**Input**	**Elicitation**	**Output-contingent**
*Parallel talk* Description of the child's or action (Teacher: “You are smiling”)	*Open-ended question* Non-specific request (Teacher: “How do you feel?”)	*Expansion* Repetition with added elements (Child: “I draw,” Teacher: “You draw a picture”)
*Repetition* Replication of the child‘s utterance (Child: “I like chocolate.”, Teacher: “Chocolate.”)	*Simple Wh-questions* Can be answered with name or label of object (Teacher: “What is that?”)	*Indirect corrective feedback* Rectification without explicit identification of the error (Child: “He see me,” Teacher: “He sees you.”)

These examples illustrate the divergence of mechanism by which the LSSs support the language development of young children. The efficacy of the strategies depends on the degree to which they match both the individual child's language proficiency and his or her immediate focus of attention (Hoff-Ginsberg, 1987; Girolametto et al., 1999). The impact of LSSs on children's language acquisition was first investigated in observational studies (e.g., Newport, 1976; Hoff-Ginsberg, 1986) with regard to the growth of expressive language (e.g., number of auxiliaries, verb or noun phrases). A few years later, a number of LSSs were combined with properties of the so-called child-directed speech^1^ (e.g., creation of a joint focus of attention) in parent- and staff-administered language interventions, such as joint book reading (Whitehurst et al., 1988, 1994; Crain-Thoreson and Dale, 1999; Hargrave and Sénéchal, 2000) or trainings that adhere to the principles of the “interactive model of language intervention” (for an overview, see Tannock and Girolametto, 1992). These interventions demonstrated facilitatory effects on the language development of young children from different socioeconomic backgrounds and different proficiency levels covering many linguistics domains (e.g., grammar, productivity, vocabulary).

However, LSSs have been studied mainly as effective pedagogical tools for language support in one-to-one or small-group interactions in a small range of activities (e.g., book reading). Transferring the use of LSSs toward large-group settings, which are common in preschool and kindergarten, has proven to be a major challenge. Whitehurst et al. (1994), for example, examined the effectiveness of LSSs integrated in a training of dialogic book reading. He found that large-group interactions, unlike dyads, exert no impact on children's language development. One reason for this may lie in the difficulties to match the LSSs with the heterogeneous levels of children's language status in larger groups (Marinac et al., 2000). Furthermore, Dickinson (2001) and Girolametto and Weitzman (2002) revealed the impact of contextual influences on extent and efficacy of LSS-usage, reminding us of the difficulty to interweave the use of LSSs with everyday conversations in physically and socially divergent settings.

For this reason, we developed the “Feeling, Thinking and Talking” (FTT)-intervention that enables teachers to create a joint conversational focus with children independent of the prevailing frame of action in order to ensure the efficacy of LSSs across different settings, including large-group interactions (e.g., circle time, free play). The FTT intervention guides teachers to initiate sustained verbal exchanges with children on their experiences related to their emotions (inner world) and the external world around them. Both topics are attractive for young children, and both are acquired through conversation. Moreover, children's verbal engagement in the discovery of the internal world of mental states and the external world that they live in benefits from the rapid development of two areas during early childhood, i.e., emotion knowledge and scientific thinking.

With this in mind, we designed the FTT-intervention to combine elements of two previous trainings in the realm of emotion knowledge and scientific thinking, namely the “Scientific Method to Guide Learning” (Gerde et al., 2013) and the “Conversational training on Preschoolers' Emotion Comprehension” (Ornaghi et al., 2015). Both conversational trainings guide teachers to initiate processes of verbal thinking about scientific phenomena (rainbows, animals etc.) as well as emotional states (desires, fears etc.) within groups of children of different ages. Furthermore, both trainings advise teachers to intensively use mental state language when describing, analyzing, and exploring observations of the antecedents and consequences of emotions and of scientific hypotheses. For example, mental state language occurs when discussing the antecedents of fear of a picture-book character and what he might think or do to alleviate this feeling or when verbalizing children's hypotheses about the origin of steam coming out of a kettle of boiling water (Tompkins et al., 2018).

However, sustained talk about emotional and scientific phenomena not only promotes the use of mental state language, but also offers the opportunity to apply LSSs. *Parallel talk*, for example, is an LSS in which the teacher comments on a child's (or her own) action and internal state by verbalizing what she is doing, thinking, or feeling (see Table 1). At the same time, it is an example of mental state language, since its communicative function is to direct the attention to the internal representation of an action, emotion, or belief, thereby assisting the child to take the perspective of the adult. Likewise, Gerde et al. (2013) propose that initial step in the “scientific method” is the description of what is being observed. Therefore, teachers should support children in labeling and expressing what they see. In other words: The “scientific method” requests the use of LSSs that provide or elicit new language input (e.g., asking simple what-questions, see Table 1). Because of this overlap of functions, many strategies used to encourage young children to reflect on their own or others' mental states also facilitate the process of language acquisition. To the best of our knowledge, the FTT intervention is the first teacher-led intervention that teaches teachers about the adaptive usage of LSSs and their application in the domains of emotion knowledge and scientific thinking.

The FTT intervention shares some of the premises and objectives of other professional trainings on early childhood teacher-child interactions, such as the CLASS-based Teachstone Trainings^2^ (Hamre et al., 2012) or the Hanen Program for Early Childhood Educators/Teachers (“Learning Language and Loving it”^3^. These commonalities notwithstanding, our intervention differs from existing ones by the integration of guidelines from two (well established) conversational trainings that serve to overcome the obstacles inherent in the application of LSSs in heterogenous group settings (as set forth above). Given the priority of this issue, the FTT intervention devotes a complete session to the topic of “transfer,” as will be shown in the following summary of the organization and sequencing of its modules.

## Content of the FTT Intervention

The FTT intervention aimed at teachers in German kindergartens, serving children from about 2 to 6 years of age, who can make use of many opportunities throughout the day and who can tailor their LSSs to individual children's language capacities. In his meta-analysis, Hattie (2009) demonstrated that teacher trainings influenced teachers' learning-related attitudes and behaviors with a medium effect size (d = 0.62). Teachers can also compensate for the lack of linguistic stimulation in the home (Vernon-Feagans et al., 2013) and training them is cost-effective provided they maintain their improved LSS with successive generations of children in their care.

The aim of the six FTT intervention modules was to teach teachers about the adaptive usage of LSSs and their application in the domains of emotion knowledge and scientific thinking. Teachers were also taught to apply the LSSs in the many everyday conversations in different settings. Module 1 targeted theories of language acquisition, in particular the acquisition of a second language, and teachers' abilities to recognize opportunities for language support. In module 2, the LSS were explained in detail and practiced in role plays. Module 3 aimed at promoting emotion knowledge by using LSS during dialogic picture-book reading and by reminiscing about shared events during meals. In module 4, teachers learned to promote scientific thinking by using LSS. Here too, dialogic picture-book reading, and mealtimes were used as training situations. Module 5 targeted extending knowledge about teachable language-support opportunities into other situations, e.g., free play. Module 6 was a refresher module on LSSs and on implementing them in everyday activities in elementary education and care institutions. The six modules were completed within 40 h. Modules 1–4 were each taught in 8 h; modules 5 and 6 were taught in 4 h each. Modules 1–5 were scheduled about a month apart to give teachers the opportunity to try out, practice, and adapt the proposed content and methods to their own teaching style. Because module 6 was a refresher module, it was scheduled three to six months after module 5. Teachers were asked to videotape their interactions with the children at three times with specific instructions. These videos were used to clarify misunderstandings and to improve teachers' LSS in one-to-one sessions with members of the research team. More detail on the FTT intervention can be found in Salisch et al. (2021).

## Evaluation of the FTT Intervention

Kirkpatrick and Kirkpatrick (2006) proposed a four-level model for the evaluation of the effectiveness of on-the-job-training programs. When applied to our FTT intervention, the first three levels refer to teachers' acceptance of the training, their learning of new concepts and attitudes, and their language-related behaviors toward the children in their care. Although a training can be effective in improving teachers' knowledge, attitudes, and behavior, the effectiveness of the intervention is ultimately determined at level four, i.e., at the level of children's language development. To this end, children whose teachers had undergone the FTT training —this was the treatment group— were compared to those whose teachers had not undergone the training—this was the business-as-usual (BAU) group—in regard to different components of their language development. Expressive and receptive vocabulary, syntax (creating morphological rules and receptive language skills), and phonological memory (for pseudo words, for sequences of words, and for sentences) were each assessed three times.

The present study focuses on the effects of the FTT intervention on the language development of the children (other papers of the research group focus on its effect on children's emotion knowledge and on their scientific thinking). Thus, the study aims to answer the following question: What is the effect of the FTT teacher training on children's language development? We expect children from the treatment group to make faster progress in objective tests of (expressive and receptive) vocabulary, morphological rules, receptive understanding of syntax, and sentence-related memory than children from the BAU group. We expect no significant differences between the two groups for the development of the memory for single words and non-words, because this basic cognitive capacity is very difficult to enhance even via direct and intensive training and exercise (Sala and Gobet, 2017; Mähler et al., 2019). Accelerated language development will be expressed statistically in a significant interaction of group (i.e., treatment vs. BAU group) over time (i.e., the three points of measurement).

## Method

### Participants

In the FTT project, data from 281 children at 13 kindergarten sites with 28 kindergarten groups in rural areas and towns in the province of Lower Saxony and in a district in Hamburg, Germany, were collected from interviews, tests, and ratings by teachers and parents. At T1 the sample size was *N* = 277 children with a mean age of 49.82 months (SD = 7.22; range = 35–66 months). The sample of children consisted of 137 (49%) girls and 143 (51%) boys, and in one case this information was missing. We collected parent questionnaires for 200 (71%) of the children. According to the combined teacher and parent report, 142 (51%) children had parents who were born in Germany, 119 (42%) children had at least one parent who was not born in Germany, and in 20 cases (7%) this information was missing. In order to document children's multilingualism, parents were asked whether their child speaks another language (apart from German), which languages are spoken in the household, and which language the parents speak best. When another language was mainly spoken in the household with the child, the child was recorded as DLL. Sixty-two children (22%) were DLL, 129 children (46%) were native German speakers, and for 90 children (32%) the information was missing due to missing parent reports. To obtain the socioeconomic status of the children's families, we used the information from the parent questionnaire. We calculated the Highest International Socio-Economic Index of Occupational Status (HISEI), which can take values between 16 and 90. It was significantly higher for the native-born German children's families (*M* = 53.39, SD = 12.78) than for the immigrant children's families [*M* = 44.43, SD = 16.11; *t*(81.82) = 3.474, *p* < 0.001]. By T2, the sample size was *N* = 238. Two children who did not participate at T1 were added to the sample, while 41 dropped out. At T3, another two children were added who did not participate at T1 or at T2, and four children participated who had left out T2. Eleven children dropped out, so that *N* = 233 (84% of T1) remained in the sample at T3. Some children were not added to the sample until T2 or T3 because they were ill or refused testing at T1. The teachers of the kindergarten groups of the treatment group and the BAU did not differ in their prior experience with language enhancement training [χ^2^(1) = 0.675, *p* = 0.411].

### Procedure

The FTT study received a positive vote of the ethics committee of the University of Hildesheim. Participation in the study was voluntary. The children were tested in a quiet room at the kindergarten sites at three measurement points. The second and third test sessions (T2 and T3) were conducted approximately six months and one year after T1, respectively. These time points were the pre-, post-, and follow up- measurements in the intervention study. Trained interviewers tested each child individually. At each measurement point, each child was tested in five blocks (max. two blocks within one day) with each block lasting for ~ 30 min. Additionally, kindergarten teachers and parents (both at T1) completed a questionnaire for each child about the socioeconomic background (parents) and the behavior of the children (teachers).

### Measures

*Expressive vocabulary*. Expressive vocabulary was measured with the revision of the Active Vocabulary Test for 3- to 5-year-old children (AWST-R; Kiese-Himmel, 2005). In the AWST-R, the child is shown a set of cards depicting objects or activities. For the 51 nouns, the interviewer asks the question “What is that?” and for the 24 verbs “What is he or she doing?” The first ten items serve as an icebreaker; if a child does not answer any of them, the test is discontinued. Correct answers represent the raw score, which can be compared to the test norms. The AWST-R norms range from 3;0 to 5;5 years. Internal consistency of the AWST-R at T1 was very high with Cronbach's α = 0.97.

*Receptive vocabulary*. The German version of the Peabody Picture Vocabulary Test (PPVT-4; Lenhard et al., 2015) was used to measure children's receptive vocabulary. The test consists of 228 items that are organized in 19 sets with an adaptive structure; there are age-related starting points as well as reversal and termination rules. Within each set, the child is shown one item consisting of four pictures. The child is asked to point to the picture that matches the word mentioned by the experimenter (“Point to…”). Each set consists of 12 items—if a child cannot point to the corresponding item for eight or more words in the set, the test is discontinued. A child can thus receive a maximum of 228 points. The PPVT-4 provides norms for children from 3;0 to 16;11 years. Internal consistency of the PPVT-4 at T1 was very high with Cronbach's α = 0.98.

*Language development*. The German language development test for 3- to 5-year-old children (SETK 3-5; Grimm, 2015) was used to assess children's general level of language development. This test consists of five subtests that measure receptive and expressive language processing skills as well as auditory memory. The data are standardized for ages ranging from 3;0 to 5;11 years.

The Understanding Sentences (US) sub-test was used to measure *sentence comprehension*. This subtest covers mainly syntax but also calls for some lexical knowledge. Children are asked to carry out the experimenter's instructions with various materials. If they do this correctly, a point is awarded. Because there are 15 tasks of increasing grammatical complexity, a total of 15 raw points can be achieved; there is no stop criterion. Internal consistency of the sub-test US at T1 was high with Cronbach's α = 0.89.

The development of *expressive grammar* was examined by means of the Morphological Rule Formation (MR) subtest. In this test, the child is shown ten picture cards with common nouns and eight picture cards with made-up nouns. The interviewer names one of the objects shown and asks the child to pronounce the name of the plural object (“This is a [noun]. […pause…] Here are [number]…?”). The test is discontinued if a child cannot name plural forms of the first ten items. In total, a maximum of 36 raw points can be achieved. Two points are awarded for each correct plural formation one point is awarded for an alternative plural formation related to the word stem. Internal consistency of the MR subtest at T1 was high with Cronbach's α = 0.91.

To test the *phonological memory for pseudo words*, the Phonological Memory for Non-Words (PMN) subtest was used. Here the child is shown a total of 18 visually represented fictitious figures (“monsters”) whose names the child is asked to repeat. When the fictitious name is correctly reproduced, a point is awarded, so that a maximum raw score of 18 points is possible. Internal consistency of the PMN subtest at T1 was good with Cronbach's α = 0.81.

The Memory Span for Word Sequences (MS) subtest is a classical span task for testing the *short-term memory for word sequences*. After a training item, the child is presented with sequences of two to a maximum of six monosyllabic words, which the child is asked to repeat in the same order. If a child fails to repeat two series of the same length, the test is terminated. In total, a raw score of ten can be achieved with ten items. Internal consistency of the MS subtest at T1 was acceptable with Cronbach's α = 0.70.

The Sentence Memory (SM) subtest is used to assess the *auditory memory for word sequences* embedded in semantic and grammatical structures. The child is asked to repeat sentences of increasing length and complexity. The first six sentences make sense in terms of content; the following nine sentences are correct only in terms of grammatical structure. A raw value point can be obtained for every correctly reproduced word; a total of 119 points are possible. The subtest is discontinued if a child fails to reproduce three meaningless sentences. Internal consistency of the SM subtest at T1 was very high with Cronbach's α = 0.95.

*Behavioral self-regulation*. Behavioral self-regulation was only included in the propensity score matching process. It was examined at T1 using the Head–Toes–Knees–Shoulders Task (HTKS; Ponitz et al., 2008). This instrument was developed for children, aged three to eight. The children were asked to do the opposite of what the experimenter did (e.g., ‘When I touch my head, you touch your toes'; ‘When I touch my toes, you touch your head'). This first rule was tested in a 10-item block. Afterwards, a second rule was introduced: touching the shoulders instead of the knees and vice versa. Adherence to both rules was tested in a second 10-trial block. In a third block, the rules of block one and two were mixed and again tested in 10 trials. The children's correct reactions (2 points) and self-corrections (1 point) were tallied (0–60 points). The test is discontinued if a child scores less than four points in a block. At 0.96, Cronbach's α was excellent.

*General cognitive abilities*. Children's general cognitive abilities were only included in the propensity score matching process. Raven's Colored Progressive Matrices (CPM; Bulheller and Häcker, 2002) were used to determine children's general cognitive abilities. Because the test is standardized for children between 45 and 140 months, we used the raw score for our statistical analyses. The children are shown 36 items and asked to nonverbally choose the missing part of a picture from a selection of six possibilities. One point is awarded for each correct answer. In the present sample, the split-half reliability was *r* = 0.61.

## Results

All statistical analyses were conducted using the software R (R Core Team, 2013). In the variables relevant for the analyses, about 17% of the data were missing. Because van Buuren and Groothuis-Oudshoorn (2011) recommended to use the amount of missing data as an indicator for the number of imputations, multiple imputation (Rubin, 1976) with 17 imputations was used to handle the missing data with the package mice. Although there were only little, non-significant differences between the treatment group and the BAU group in the variables age, socioeconomic status, immigrant status, multilingualism, behavioral self-regulation, and general cognitive abilities, the data were matched using nearest neighbor matching with the package MatchIt (Ho et al., 2011). Balance in covariates was ascertained by checking the distribution of propensity scores of both treatment and matched control units, as well as inspecting their mean difference before and after matching with the packages MatchIt (Ho et al., 2011) and Cobalt (Greifer, 2020). Matched units were well-balanced with respect to all covariates (results are provided in the Figures 1, 2 in the Supplementary Material). The Chi-square test of mean differences after matching was non-significant for all covariates [χ^2^(6) = 7.22, *p* = 0.301]. Due to the matching procedure, the actual sample size in each of the imputed data sets after the matching varied between 216 and 266.

For the longitudinal data, multilevel mixed effects models were calculated using the nlme package (Pinheiro et al., 2020). A three-level model was designed for each language variable. The children's scores at the three measurement points represented level one and were nested in the children (level two). The children were nested in the kindergarten groups, which formed level three. In the analysis, 216–266 children in 28 kindergarten groups led to 648–798 observations.

Table 2 shows the bivariate Pearson correlations between child characteristics and outcome variables at T1 with the original data. According to Cohen's convention (Cohen, 1988), all language variables correlated positively with each other at medium to high levels. The children's age did not correlate with the HISEI and MR. The HISEI correlated positively with all language variables at a medium level. T-tests showed that there were no significant differences between boys and girls for any of the language variables. However, DLLs showed a significantly lower mean score in almost all language variables than monolingual German children (see Supplementary Table 1). Therefore, multilingualism was included, and gender was not controlled for in the following analyses.

**Table 2:** Descriptive statistics and bivariate Bonferroni-corrected Pearson correlations (*N*) of children's characteristics and relevant variables at T1.

	**HISEI**	**Age**	**PPVT**	**AWST**	**US**	**SM**	**PMN**	**MR**	***N***	**Mean (SD)**	**Range**	**Skew**	**Kurtosis**
HISEI									155	50.45 (14.54)	18–65	−0.63	−0.77
Age	−0.07 (153)								277	49.82 (7.22)	35–66	0.14	−0.96
PPVT	0.34^***^ (148)	0.30^***^ (266)							266	65.62 (31.41)	1–133	−0.18	−0.57
AWST	0.38^***^ (146)	0.16^**^ (254)	0.85^***^ (246)						254	24.83 (16.01)	0–60	0.00	−1.04
US	0.45^***^ (149)	0.18^**^ (250)	0.72^***^ (242)	0.73^***^ (239)					250	6.93 (4.45)	0–15	−0.12	−1.32
SM	0.40^***^ (122)	0.15^*^ (203)	0.70^***^ (198)	0.72^***^ (196)	0.72^***^ (196)				203	56.69 (29.61)	2–117	0.02	−1.00
PMN	0.31^**^ (146)	0.30^***^ (256)	0.57^***^ (249)	0.56^***^ (244)	0.51^***^ (242)	0.58^***^ (201)			256	6.64 (3.92)	0–16	0.10	−0.73
MR	0.33^***^ (141)	0.09 (240)	0.65^***^ (233)	0.69^***^ (229)	0.65^***^ (229)	0.60^***^ (195)	0.44^***^ (236)		240	15.18 (9.90)	0–32	−0.14	−1.33
MS	0.22^*^ (148)	0.28^***^ (255)	0.43^***^ (250)	0.40^***^ (242)	0.44^***^ (241)	0.62^***^ (201)	0.51^***^ (251)	0.36^***^ (236)	255	4.19 (1.63)	0–8	−0.15	0.27

*** *p < 0.001*;

** *p < 0.01*;

* *p < 0.05*;

*PPVT, Peabody Picture Vocabulary Test; AWST, Active Vocabulary Test; Us: Understanding Sentences; MR, Morphological Rule Formation; PMN, Phonological Memory For Non-Words; MS, Memory Span For Word Sequences; SM, Sentence Memory*.

When measuring changes in the different language variables with multi-level models in the treatment and the BAU group, the children's age at the first measurement point, their family's socioeconomic status (HISEI), and their multilingualism were controlled.

Table 3 indicates that all children's performance increased markedly over time for all language variables. Children's age at the first measurement point was a predictor of their performance for all language variables, with older children scoring higher. Growing up in a multilingual family had a significantly negative impact on all language variables except for PMN and MS, whereas a higher HISEI predicted a better performance on all variables. The children's initial language scores were not affected by whether their teachers belonged to the treatment group or the BAU group.

**Table 3:** Effects of FTT on all language skills over time controlled for age, multilingualism, and HISEI.

	**Receptive vocabulary**					**Expressive vocabulary**									
	**β**	**SE**	**df**	***T***	***f*^2^**	**β**	**SE**	**df**	***t***	***f*^2^**					
Intercept	−0.563	0.144	328.315	−3.897^***^	−0.449	0.122	345.479	−3.669^***^							
Time	0.370	0.032	154.592	11.647^***^	0.37	0.342	0.021	191.890	16.190^***^	0.61					
Group (TG = 1)	0.008	0.189	375.878	0.043	0.02	0.012	0.158	383.330	0.073	0.02					
Age (T1)	0.389	0.042	123.588	9.157^***^	0.27	−0.758	0.096	122.404	−7.908^***^	0.10					
Multilingualism	−0.640	0.098	86.681	−6.509^***^	0.28	0.248	0.045	99.497	5.483^***^	0.40					
HISEI	0.285	0.065	32.032	4.383^***^	0.20	0.268	0.061	39.933	4.386^**^	0.21					
Group*Time	0.069	0.043	223.969	1.609	0.00	0.058	0.031	130.813	1.846	0.01				
	**Sentence understanding**					**Morphological rule formation**									
	**β**	**SE**	**df**	***T***	***f***^2^	**β**	**SE**	**df**	***t***	***f***^2^					
Intercept	−0.279	0.136	306.767	−2.046^*^	−0.184	0.152	343.791	−1.211							
Time	0.247	0.043	139.473	5.752^***^	0.14	0.189	0.043	193.730	4.413^***^	0.14					
Group (TG = 1)	−0.248	0.182	268.563	−1.358	0.01	−0.238	0.202	326.297	−1.178	0.06					
Age (T1)	0.282	0.048	124.411	5.870^***^	0.12	0.223	0.053	100.029	4.207^***^	0.11					
Multilingualism	−0.555	0.106	123.660	−5.259^***^	0.18	−0.540	0.121	81.152	−4.478^***^	0.21					
HISEI	0.311	0.081	26.804	3.855^**^	0.22	0.292	0.076	34.968	3.850^**^	0.22					
Group*Time	0.117	0.060	158.196	1.971^*^	0.01	0.128	0.064	124.192	2.004^*^	0.06					
	**Phonological memory for non-words**					**Sentence memory**					**Memory for word sequences**				
	**β**	**SE**	**df**	***T***	***f***^2^	**β**	**SE**	**df**	***t***	***f***^2^	**β**	**SE**	**df**	***t***	***f***^2^
Intercept	−0.413	0.151	210.698	−2.731^**^		−0.431	0.132	335.777	−3.261^**^		−0.330	0.166	194.820	−1.989^*^	
Time	0.225	0.051	250.018	4.411^***^	0.09	0.292	0.033	215.721	8.867^***^	0.28	0.226	0.057	78.377	3.979^***^	0.03
Group (TG = 1)	−0.224	0.191	325.744	−1.175	0.01	−0.267	0.176	295.324	−1.518	0.01	−0.011	0.214	244.558	−0.052	0.01
Age (T1)	0.337	0.052	184.493	6.454^***^	0.14	0.264	0.051	92.329	5.145^***^	0.09	0.323	0.058	112.218	5.606^***^	0.12
Multilingualism	−0.169	0.136	64.289	−1.248	0.02	−0.446	0.105	147.425	−4.257^***^	0.14	−0.138	0.118	197.072	−1.171	0.01
HISEI	0.308	0.072	49.747	4.265^**^	0.09	0.323	0.069	39.212	4.681^***^	0.21	0.219	0.078	42.923	2.792^**^	0.05
Group*Time	0.143	0.074	193.336	1.949	0.00	0.140	0.051	99.829	2.715^**^	0.01	−0.068	0.075	119.354	−0.905	0.00

*** *p < 0.001*;

** *p < 0.01*;

* *p < 0.05; p < 0.10; TG = treatment group*.

The interaction effect between FTT and time was significant for the US, MR, and SM variables. That is, children whose teachers had undergone the FTT training demonstrated greater proficiency in understanding sentences, morphological rule formation, and sentence memory over time than their age-mates from the BAU group (β = 0.117, *p* = 0.049; β = 0.128, *p* = 0.047; β = 0.140, *p* = 0.008, respectively). According to Cohen (1992) effect sizes for all significant interaction effects were small (*f*^2^ ≤ 0.06). The interaction effects between FTT and time on the Expressive Vocabulary (β = 0.058, *p* = 0.067) and Phonological Memory for Non-Words (β = 0.143, *p* = 0.053) were marginally significant. The children in the treatment group showed a trend to be more proficient in expressive vocabulary and phonological memory for pseudo-words over time than the children in the BAU group. There was no significant group^*^time interaction for Receptive Vocabulary and for Memory Span for Word Sequences.

Intraclass correlations (ICCs) were calculated for each multilevel model to indicate the proportion of variance explained by the random effects at the different levels. The ICCs are presented in Table 4. The proportion of variance explained at the kindergarten group level is higher for the PPVT and AWST (29 and 28%, respectively) than for the other variables (≤ 17%). At the individual level, however, the proportion of variance explained is ≥ 50% for all variables.

**Table 4:** ICCs of random effects at kindergarten group level, individual level, and both levels for each outcome variable.

**Level**	**PPVT**	**AWST**	**US**	**SM**	**PMN**	**MR**	**MS**
Kindergarten group	0.29	0.28	0.11	0.12	0.08	0.17	0.09
Individual	0.50	0.59	0.70	0.66	0.60	0.56	0.51
Kindergarten group/individual	0.79	0.86	0.81	0.78	0.68	0.73	0.61

*PPVT, Peabody Picture Vocabulary Test; AWST, Active Vocabulary Test; US, Understanding Sentences; MR, Morphological Rule Formation; PMN, Phonological Memory For Non-Words; MS, Memory Span For Word Sequences; SM, Sentence Memory*.

## Discussion

The aim of the FTT professional education was to sensitize teachers in early childhood education to their own use of language while talking with the children about numerous topics, and to the integration of language support into this talk by using LSS in a variety of settings. Using customized, activity-appropriate language adapted to mealtimes, changing clothes, free play, etc., while simultaneously engaging with the individual needs of each child, is a challenge. Additionally, LSS practitioners need to be in tune with emotional or factual topics and provide the right measure of support for children to develop their own language and own ideas. This is even more difficult when addressing more than one child in group settings, because children tend to be at different levels of language proficiency. Despite these challenges, the FTT intervention with teachers seems to have succeeded in accelerating the language development of the children in their care.

The evaluation results of the fixed effects in the multilevel models indicate that the children in the treatment group made greater progress in more complex language processing skills. They outperformed the children in the BAU group regarding the three indicators: morphological rule formation, sentence comprehension and sentence memory. That is, children whose teachers received the FTT training made faster progress in their application of the complex rules of German plural formation, in their ability to understand sentences and to carry out instructions of increasing grammatical complexity, and in their memory for sentences. The latter is particularly encouraging, as sentence memory has been shown to be the strongest predictor of written language performance in primary school (Goldammer et al., 2010). These results are further supported by the variance explained at the random effects level. They show that for these three variables a relatively high proportion of the variance is explained at the individual level. This fits well with the FTT intervention, in which LSS (and other verbal utterances) are adjusted to individual children's language skills. Together, these complex processing abilities are part of the register of academic language, which seems to contribute most to children's educational success (Volodina et al., 2020).

An explanation of these effects could be the longer conversations that provide more opportunities for teachers to model correct language use, elicit child speech, and give corrective feedback. When observing the teachers on videotape, we were able to establish that trained teachers' use of modeling techniques was related to longer dialogues with more turn-taking, which included a higher number of turns provided by the children (Hormann et al., 2021). These and other mediating mechanisms need to be followed up in the future.

The positive effects of the FTT teacher intervention on children's language skills were established after important covariates, such as age, SES, and growing up in a multilingual family, had been considered. This is interesting because the covariates seemed to exert a significant influence on children's language acquisition. The main effects underline the well-known influences of SES (e.g., Rowe, 2018) and DLL on young children's language development (e.g., Volodina et al., 2020) and extend them to a heterogeneous sample of young children in Germany. As in many other countries, children growing up in low-SES families scored lower on all language measures. In contrast to other studies, this was true even for measures of short-term memory (e.g., Engel et al., 2008; Alloway et al., 2014). DLLs showed a smaller receptive and expressive vocabulary, lower grammatical abilities, and a somewhat lower memory for sentences throughout the study, even after their SES had been controlled for. The same is true for short-term-memory for pseudo words and the measure for word sequences shows a similar tendency, which supports the notion that short-term memory is a very basic cognitive capacity. The DLLs' learning curves in all areas of language, however, did not differ from those of their monolingual age-mates. This suggests that the FTT intervention benefits the complex language processing skills of all children, including the DLLs.

All things considered, the study results underscore the importance of improving teacher-child dialogues by motivating teachers to use LSS and to set up teaching opportunities for utilizing these strategies because they model language input, elicit child language, and provide feedback to child language in authentic situations of joint attention which are best suited for child language learning (Vygotsky, 1962). Teachers in early childhood education who were trained to use this variant of child-directed speech seem to be able to foster children's development of complex language processing skills during their day-to-day activities in a variety of settings. Because these language skills are part of the register of academic language, the FTT intervention served to enhance children's readiness for formal schooling.

The FTT teacher intervention did not show any significant effects (either no effect or marginal trends) on children's receptive (or expressive) vocabulary. Integrating language promotion into everyday life activities seems to be less suitable for broadening children's vocabulary. Instead, this may depend less on lengthy supportive conversations than on exchanges on specific topics of interest to the children. Moreover, the ICCs showed that about a third of the variance in vocabulary are explained at the kindergarten group level. This suggests that group-level variables that cannot be influenced by the intervention (e.g., kindergarten neighborhood and associated SES) also influenced children's vocabulary. As expected, we found no significant effect of the intervention on the development of the short-term memory for word spans or non-words because, as mentioned before, this basic cognitive capacity is very difficult to enhance (Sala and Gobet, 2017; Mähler et al., 2019).

## Strengths and Limitations

The FTT intervention combines a teacher training on LSS with the application in various group settings and topics of teacher-child communication (emotion talk and scientific thinking). The strengths of our study include the transfer of the effects of the FTT teacher training on the children in their groups, which was examined in a large and heterogeneous sample of 3-to-6-year-olds from 13 kindergarten sites. Noteworthy too, is the high proportion (42%) of children from immigrant families, which corresponds to their number in representative samples (e.g., Bock-Famulla et al., 2018). Strong points also include children's nesting within their kindergarten groups which controls for institutional effects, teaching styles, and group composition effects. The fact that the children in the treatment group and those in the BAU group did not differ in terms of their initial language abilities underlines the robustness of the intervention results and constitutes another strength of this study.

Yet, some limitations of the study must be mentioned. Unfortunately, it was not possible to randomize the participating centers into the treatment and the BAU group. This was alleviated with the propensity matching procedure. The FTT program was taught at the various institutions with a detailed manual. Nevertheless, deviations in teaching due to different trainers cannot be ruled out. Finally, we could only report mean level effects on the level of the children which include large variances and high overlaps between the intervention and the BAU group.

## Outlook

Future studies should be devoted to the mechanism of change. How often teachers talk to children and how often they use specific LSSs could mediate the effects on the language development of the children in their groups. Teachers' liking of the strategies and their enthusiasm when using them could be further mediators. Further analyses should examine whether the effects found are comparable in all 13 institutions of early childhood education, or whether group effects and staffing ratios impact on the results of the FTT intervention. And, finally yet importantly, some effects of the FTT intervention may not be immediate. They may only surface after a prolonged exposure to teachers' improved LSS. A long-term follow-up in primary school is needed.

## Conclusions

The results of this study highlight the potential of using LSS in kindergarten settings in order to further children's language development and later school careers. Training teachers to use such strategies represents an important component of teachers' training. Kindergarten teachers act as multipliers and the use of LSS can have positive effects on multiple generations of children. Future study should focus on possible moderators and mediators of the effects and investigate long-term effects.

## Data Availability Statement

The raw data supporting the conclusions of this article will be made available by the authors, without undue reservation.

## Ethics Statement

The studies involving human participants were reviewed and approved by Ethics Committee of the University of Hildesheim, Germany. Written informed consent to participate in this study was provided by the participants' legal guardian/next of kin.

## Author Contributions

KV did all analyses, wrote the main part of the method section with figures and tables, did the proofreading, and cleansing of text. OH wrote introduction about language support strategies and language interventions. MP helped with analyses and helped writing the part about propensity score matching. CM wrote discussion and did proofreading. MS wrote introduction and discussion. All authors contributed to the article and approved the submitted version.

## Conflict of Interest

The authors declare that the research was conducted in the absence of any commercial or financial relationships that could be construed as a potential conflict of interest.

## Footnotes

^1^Child-directed speech is characterized by a fine-tuning of themes, speed, prosody and complexity to the capacities of infants or young children (Snow, 1972; Newport et al., 1977).

^2^https://teachstone.com/

^3^http://www.hanen.org/Programs/For-Educators/Learning-Language-Loving-It.aspx)
