# Supplementary material for: Teaching the Teachers About Language Support Strategies: Effects on Young Children's Language Development
Source: Front Psychol. 2021 May 4;12:660750. doi: 10.3389/fpsyg.2021.660750 (PMC8129529; doi:10.3389/fpsyg.2021.660750)
Supplement: Supplementary file 1 [file Table_1.docx]

Table 1. Mean differences of the language variables for gender and multilingualism at T1

|  | Mean (SD) | |  | Mean (SD) | |  |  |
| --- | --- | --- | --- | --- | --- | --- | --- |
|  | Boys | Girls | t | Monolingual | DLL | t | |
| PPVT | 68.30 (31.20) | 63.15 (31.52) | 1.336 | 82.30 (24.51) | 50.93 (28.83) | 7.208*** | |
| AWST | 25.01 (15.19) | 24.66 (16.84) | 0.175 | 34.10 (12.55) | 16.32 (13.86) | 8.199*** | |
| US | 7.26 (4.48) | 6.63 (4.40) | 1.117 | 8.70 (3.81) | 4.98 (4.23) | 5.630*** | |
| MR | 15.86 (10.40) | 14.63 (9.33) | 0.958 | 19.35 (8.18) | 11.21 (9.54) | 5.356*** | |
| PMN | 6.71 (3.78) | 6.54 (4.06) | 0.349 | 7.35 (3.79) | 5.66 (3.71) | 2.805** | |
| MS | 4.33 (1.62) | 4.05 (1.65) | 1.356 | 4.39 (1.55) | 3.93 (1.79) | 1.677˙ | |
| SM | 72.99 (31.28) | 67.23 (28.91) | 0.587 | 67.80 (27.86) | 46.51 (26.75) | 4.123*** | |
| *Note*. ***: p < .001; **: p < .01; *: p < .05; ˙: p < .10; Mono: Monolingual, Multi: Multilingual; PPVT: Peabody Picture Vocabulary Test; AWST: Active Vocabulary Test; US: Understanding Sentences; MR: Morphological Rule Formation; PMN: Phonological Memory for Non-Words; MS: Memory Span for Word Sequences; SM: Sentence Memory | | | | | | | |
