# Supplementary figures and images for: Teaching the Teachers About Language Support Strategies: Effects on Young Children's Language Development
Source: Front Psychol. 2021 May 4;12:660750. doi: 10.3389/fpsyg.2021.660750 (PMC8129529; doi:10.3389/fpsyg.2021.660750)

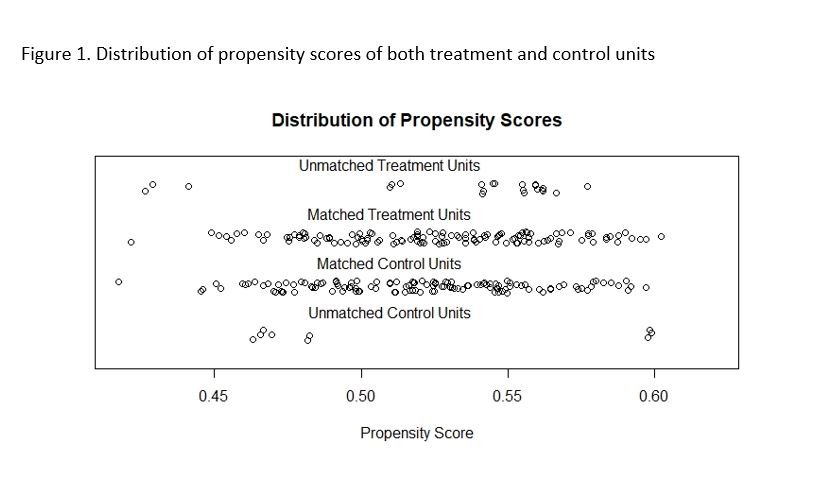

Supplement: Supplementary file 2 [file Image_1.jpg]

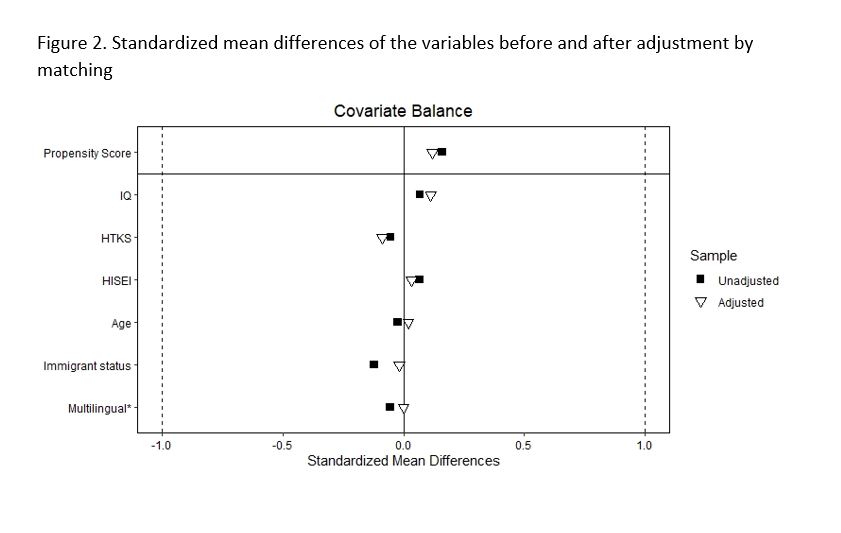

Supplement: Supplementary file 3 [file Image_2.jpg]
